# Optimization of Posterior Fossa Image Quality Using Virtual Monoenergetic Reconstructions from Dual-Layer Spectral CT

**DOI:** 10.3390/tomography12090120

**Published:** 2026-08-24

**Authors:** Helena Mellander Oxholm, Veronica Fransson, Björn M. Hansen, Birgitta Ramgren, Kristina Ydström, Teresa Ullberg, Johan Wassélius

**Affiliations:** 1 Stroke Imaging Research Group, Department of Clinical Sciences, Lund University, 22185 Lund, Sweden; helena.mellander_oxholm@med.lu.se (H.M.O.); bjorn.hansen@med.lu.se (B.M.H.); birgitta.ramgren@med.lu.se (B.R.); teresa.ullberg@med.lu.se (T.U.); 2 Department of Radiology, Skåne University Hospital, 22185 Lund, Sweden; 3 Department of Translational Medicine, Medical Radiation Physics, Lund University, 20502 Malmö, Sweden; veronica.fransson@med.lu.se (V.F.); kristina.ydstrom@med.lu.se (K.Y.); 4 Radiation Physics, Department of Hematology, Oncology and Radiation Physics, Skåne University Hospital, 22185 Lund, Sweden; 5 Department of Neurology, Skåne University Hospital, 20502 Malmö, Sweden

**Keywords:** dual-layer spectral CT, virtual monoenergetic images, posterior fossa, beam-hardening artifacts, image quality, image optimization, non-contrast head CT

## Abstract

**Background/Objectives**: Beam-hardening artifacts from the skull base reduce image quality in routine non-contrast head CT, particularly in the posterior fossa. Virtual monoenergetic images (VMIs) reconstructed from dual-layer spectral CT have been shown to reduce these artifacts, but previous studies have generally been limited by small cohorts or evaluation of only a limited range of monoenergetic reconstructions. The aim of this study was to comprehensively evaluate image quality and posterior fossa artifact reduction across the monoenergetic spectrum in a large cohort of normal head CT examinations. **Methods**: Consecutive adult patients undergoing non-contrast head CT on a dual-layer spectral CT system were retrospectively included if no intracranial pathology was identified on clinical interpretation. Regions of interest were placed in predefined posterior fossa and supratentorial locations in conventional images and automatically propagated to VMIs reconstructed from 40 to 200 keV. Image noise, signal-to-noise ratio (SNR), contrast-to-noise ratio (CNR), attenuation, and a posterior fossa artifact index were quantified. Two neuroradiologists independently performed qualitative image quality assessment. **Results**: A total of 188 examinations were included in the quantitative analysis and 40 in the qualitative assessment. Compared with conventional images, VMIs reconstructed at ≥50 keV demonstrated significantly reduced image noise in the posterior fossa together with a higher SNR, while the CNR was improved between 40 and 80 keV. Qualitative assessment likewise demonstrated superior overall image quality and reduced artifact severity, with the highest ratings generally obtained for reconstructions around 60 keV. Improvements became marginal above approximately 80 keV. **Conclusions**: Virtual monoenergetic reconstructions from dual-layer spectral CT improve posterior fossa image quality by reducing beam-hardening artifacts while maintaining favorable image noise and tissue contrast. Intermediate-energy reconstructions (approximately 50–70 keV) provide the most favorable balance between beam-hardening artifact reduction, image noise, and tissue contrast, supporting their use as an image optimization strategy for non-contrast head CT. Whether these technical improvements translate into improved diagnostic performance should be evaluated in future studies including patients with posterior fossa pathology.

## Simple Summary

Beam-hardening artifacts from the skull base reduce the quality of head CT examinations and make evaluation of the posterior fossa more difficult. Spectral CT enables reconstruction of virtual monoenergetic images that may reduce artifacts without additional radiation exposure or repeat scanning. In this study, we evaluated image quality in a large cohort of normal head CT examinations and found that virtual monoenergetic reconstructions, particularly around 60 keV, reduced posterior fossa artifacts while improving both objective and subjective image quality. These findings support the use of spectral CT as a practical image optimization technique and provide a basis for future studies evaluating its impact on diagnostic performance.

## 1. Introduction

Diagnostic assessment of the posterior fossa using non-contrast head computed tomography (CT) remains challenging because of beam-hardening artifacts originating from the dense skull base. These artifacts arise when lower-energy photons are preferentially absorbed by the petrous temporal bones, resulting in distortion of attenuation values, increased image noise, and reduced grey–white matter differentiation in the posterior fossa [1,2]. The resulting degradation of image quality may limit visualization of the brainstem and cerebellum, particularly in routine emergency CT examinations.

Although magnetic resonance imaging (MRI) provides superior assessment of posterior fossa pathology, including ischemia, inflammatory disease, and neoplasms, MRI is often unavailable in the acute setting, requires substantially longer examination times, and may be contraindicated in some patients [3,4]. Consequently, optimizing the image quality of routine head CT remains clinically relevant, even when MRI is ultimately performed.

Dual-energy CT (DECT) enables material-specific image reconstruction and generation of virtual monoenergetic images (VMIs), which simulate images acquired using monochromatic X-ray beams at selectable energy levels. Depending on the acquisition technology—including rapid kV switching, dual-source systems, or dual-layer detector CT—VMIs can reduce beam-hardening artifacts while simultaneously influencing image noise, tissue contrast, and contrast-to-noise ratio [5,6]. Dual-layer spectral CT is particularly attractive because spectral information is acquired simultaneously without changes to routine scanning protocols or additional radiation exposure [7].

Previous studies have demonstrated that VMIs improve image quality in unenhanced head CT, including reduced posterior fossa artifacts and improved grey–white matter differentiation [8,9,10]. However, these studies have generally been limited by relatively small patient cohorts, evaluation of a restricted number of monoenergetic reconstructions, or both. Consequently, the optimal reconstruction energy and the robustness of these findings across a larger patient population remain incompletely established.

Accordingly, we performed a comprehensive quantitative and qualitative evaluation of posterior fossa image quality across the monoenergetic reconstruction spectrum using dual-layer spectral CT in a large cohort of normal non-contrast head CT examinations. By isolating the effect of reconstruction energy in examinations without intracranial pathology, we aimed to characterize how VMIs influence beam-hardening artifacts, image noise, signal-to-noise ratio, contrast-to-noise ratio, and subjective image quality, thereby providing further evidence to guide image optimization in spectral head CT.

## 2. Materials and Methods

### 2.1. Study Population

We retrospectively included consecutive adult patients (≥18 years) who underwent non-contrast head CT using a dual-layer spectral CT scanner (IQon Spectral CT, Philips Healthcare, Best, The Netherlands) between 1 October and 30 November 2018. Only examinations reported as normal, without evidence of ischemia, intracranial hemorrhage, mass lesion, or other structural pathology, were included.

Restricting the study population to examinations without intracranial pathology allowed for assessment of reconstruction-dependent changes in attenuation, image noise, and beam-hardening artifacts without confounding from lesion-related alterations in tissue attenuation or image interpretation.

The Swedish Ethical Review Authority approved the study and waived individual informed consent (reference number 2019-02225).

Generative AI (ChatGPT version 1.2026183, OpenAI Inc., San Francisco, CA, USA) was used for language editing. All edits were reviewed and approved by the authors.

### 2.2. Image Acquisition and Reconstruction Parameters

All examinations were acquired using the institutional standard non-contrast head CT protocol: tube voltage 120 kilovolt (kV), tube current modulation (DoseRight, DoseRight Index 37, Philips Healthcare, Best, The Netherlands), collimation 64 × 0.625, pitch 0.36 and rotation time 0.33 s. The dose length product (DLP) was noted for each patient, and the effective dose was calculated by multiplying DLP with a conversion factor of 0.0024 mSv/mGycm [11] to characterize the radiation exposure associated with the acquisition protocol.

For this study, the spectral image file for each patient was then transferred to IntelliSpace Portal (ISP) (Philips Healthcare, Best, The Netherlands) v. 10.1.4.21403 software, where axial CIs of 4 mm slice thickness, as per local routine, were reconstructed and all subsequent image measurements performed.

### 2.3. Quantitative Analysis Method

Quantitative image analysis was performed using standardized regions of interest (ROIs) positioned in predefined posterior fossa and supratentorial reference locations to evaluate both local artifact behavior and unaffected reference tissue.

Five predefined circular ROIs (10 ± 1 mm diameter) were manually positioned on the coventional image series by a single observer using standardized anatomical landmarks. The ROIs were subsequently propagated automatically by the spectral analysis software to all monoenergetic reconstructions, ensuring identical anatomical sampling across all energy levels. These ROIs were as follows:

- cerebellar grey matter,
- cerebellar white matter,
- the interpetrous portion of the pons,
- thalamic grey matter (reference), and
- supratentorial white matter (reference).

**Figure 1:**
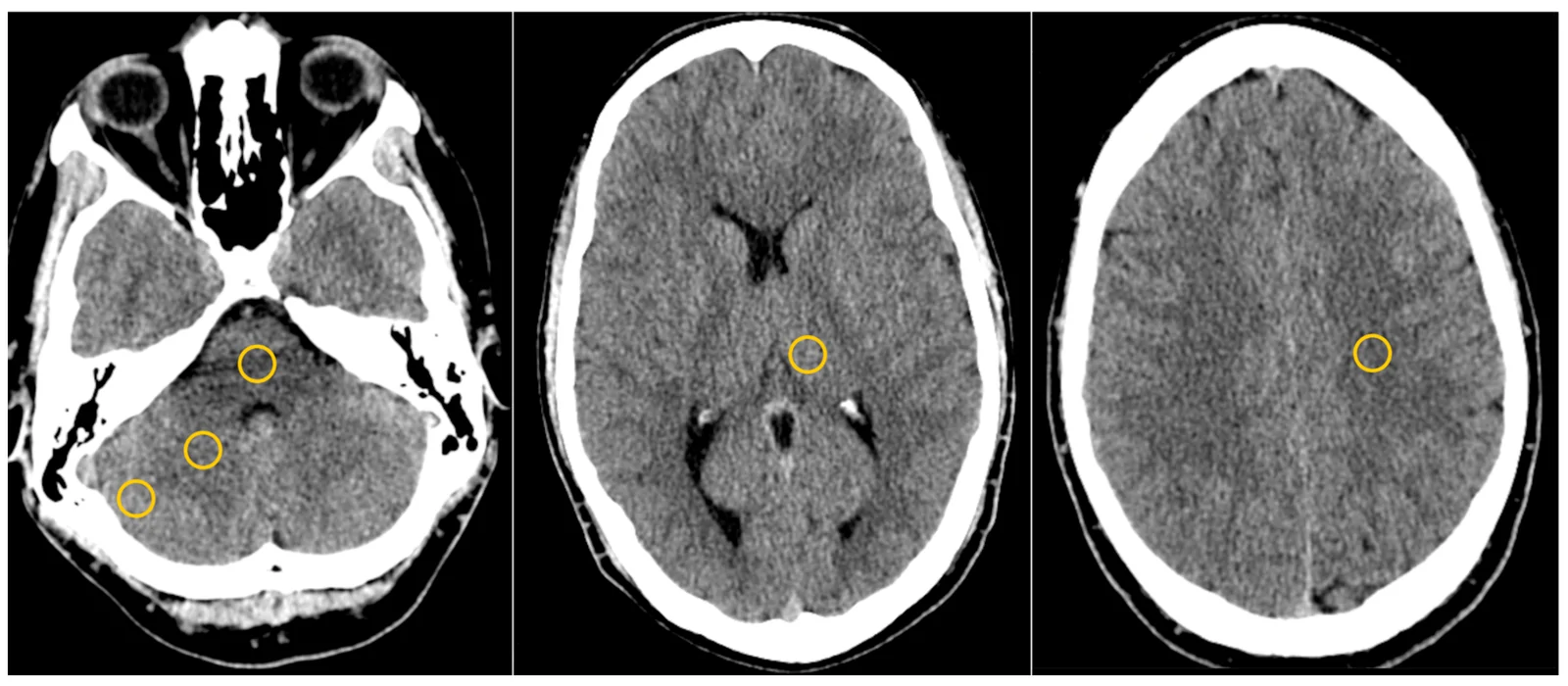
Example of ROI placement (yellow circles) in the interpetrous pons, cerebellar grey–white matter (**left** image), reference thalamic grey matter (**middle**) and reference white matter (**right**).

Reference ROIs were included to distinguish reconstruction-related image changes specific to the posterior fossa from global changes in image characteristics (Figure 1).

Mean and standard deviation (SD) attenuation values measured in Hounsfield units (HU) were noted for each ROI in the CIs and retrieved through spectral diagrams using the ISP software for the corresponding ROIs in VMIs at 40–200 keV in 10 keV intervals.

The standard deviation of attenuation within each ROI was used as a surrogate measure of image noise. The SD of the ROI in the interpetrous pons was used as a surrogate measure of beam-hardening artifact severity (Posterior Fossa Artifact Index, PFAI), in accordance with previous studies [8,9].

The SNR was calculated using previously described formulas HU/SD where HU indicates the mean attenuation of the tissue in Hounsfield units and SD the standard deviation (image noise) of the tissue within the ROI.

The contrast-to-noise ratio (CNR) was calculated for reference GM–WM and cerebellar GM–WM as (HU^1^ − HU^2^)/√ (SD^1^ + SD^2^), where HU_1 and HU_2 represent the mean attenuation of the two tissues and SD_1 and SD_2 their corresponding standard deviations. This CNR formulation has been used in previous spectral head CT studies [12,13] and was selected to maintain methodological comparability with previous work. The difference in mean attenuation (ΔHU) was calculated for cerebellar WM–interpetrous pons as previously described [10].

### 2.4. Qualitative Analysis Method

Forty consecutive examinations from the study cohort were included in the qualitative analysis. Conventional images (CIs) and virtual monoenergetic images (VMIs) reconstructed at 40–200 keV (10 keV increments) were independently reviewed by two interventional neuroradiologists with 20 and 8 years of experience, respectively. Only Reviewer 1 repeated the qualitative assessment after a three-week washout period; therefore, intra-reader agreement was calculated only for Reviewer 1.

The readers were blinded to the reconstruction energy level and independently evaluated all image series. Before the independent assessments, both reviewers jointly calibrated the scoring system using image sets not included in the study. The individual assessment and grading were performed using Viewdex (Viewer for Digital Evaluation of X-ray images) version 3.0 software [14], which allows for visualization of an axial CT stack with standard window settings (width = 65, level = 35). The readers were allowed to adjust window settings. VMIs and CIs were randomly presented to the reviewers for grading using the Viewdex software. All images were displayed on standard diagnostic monitors (Coronis^®^ Fusion MDCC-6430 6MP, Barco, Kortrijk, Belgium). CIs and VMIs at 40, 50, 60, 70, 80, 100, 120, 140 and 200 keV were included in the qualitative analysis based on both previous work [8,9,10] and visual assessment of the graphs of the quantitative part of this study.

Images were assessed using a five-point Likert scale for:

- overall image quality;
- grey–white matter differentiation in the cerebellum;
- grey–white matter differentiation in the supratentorial brain;
- severity of artifacts in the temporal cerebellum;
- severity of artifacts in the interpetrous pons; and
- severity of supratentorial subcalvarial artifacts.

In addition, the readers recorded whether they considered each image series to provide sufficient image quality to allow for confident exclusion of major posterior fossa or supratentorial pathology. This assessment reflected subjective reader confidence rather than diagnostic accuracy because only examinations without intracranial pathology were included.

Higher scores indicated better image quality, improved grey–white matter differentiation, and less severe artifacts.

### 2.5. Statistical Analysis

Continuous data are presented as mean ± SD, or mean with 95% confidence interval, and nominal and ordinal data as mean ± SD or quantity (%). A paired sample t-test was used to compare paired, parametric, continuous data (values of the same ROI but different reconstructions) and an unpaired sample t-test compared unpaired, parametric, continuous data (for example comparing values of different ROIs or CNR for reference/cerebellar tissue). A Wilcoxon signed rank test was used to compare paired ordinal data (such as values from the same task of the subjective analysis but for different reconstructions), assuming equal steps within the ranking scale. *p* values ≤0.05 were considered statistically significant. Because the analyses were primarily exploratory and aimed to characterize reconstruction-dependent changes across the monoenergetic spectrum, no formal adjustment for multiple comparisons was applied. Results should therefore be interpreted with emphasis on the magnitude, consistency, and biological plausibility of the observed effects rather than isolated *p*-values. Statistical calculations were performed using IBM SPSS Statistics for Windows, version 28.0 (IBM Corp., Armonk, NY, USA). Intra- and interrater agreement was calculated as a weighted Cohen’s kappa coefficient using MedCalc v. 22.023 software (MedCalc, Ostend, Belgium) and interpreted as suggested by Altman [15].

## 3. Results

### 3.1. Study Population Characteristics

A total of 188 patients were included in the quantitative analysis, of whom 40 consecutive patients were included in the qualitative analysis. The mean age was 62 ± 21 years in the overall cohort and 63 ± 22 years in the qualitative cohort. Women accounted for 59% (111/188) of the overall cohort and 60% (24/40) of the qualitative cohort. The mean effective radiation dose was 1.8 ± 0.3 mSv for all examinations and 1.7 ± 0.2 mSv for those included in the qualitative analysis.

### 3.2. Quantitative Analysis

#### 3.2.1. Attenuation Values

Mean attenuation values in thalamic grey matter, supratentorial white matter, and cerebellar grey–white matter decreased with increasing VMI energy, with the largest changes observed below approximately 100 keV. Mean attenuation in thalamic and cerebellar grey matter was significantly higher in VMIs at 40, 50, and 60 keV than in CIs, although the differences at 50 and 60 keV were small. In supratentorial white matter, attenuation was significantly higher in VMIs at 40 and 50 keV than in CIs. In contrast, attenuation in cerebellar white matter was significantly lower in VMIs ≥ 50 keV than in CIs.

**Figure 2:**
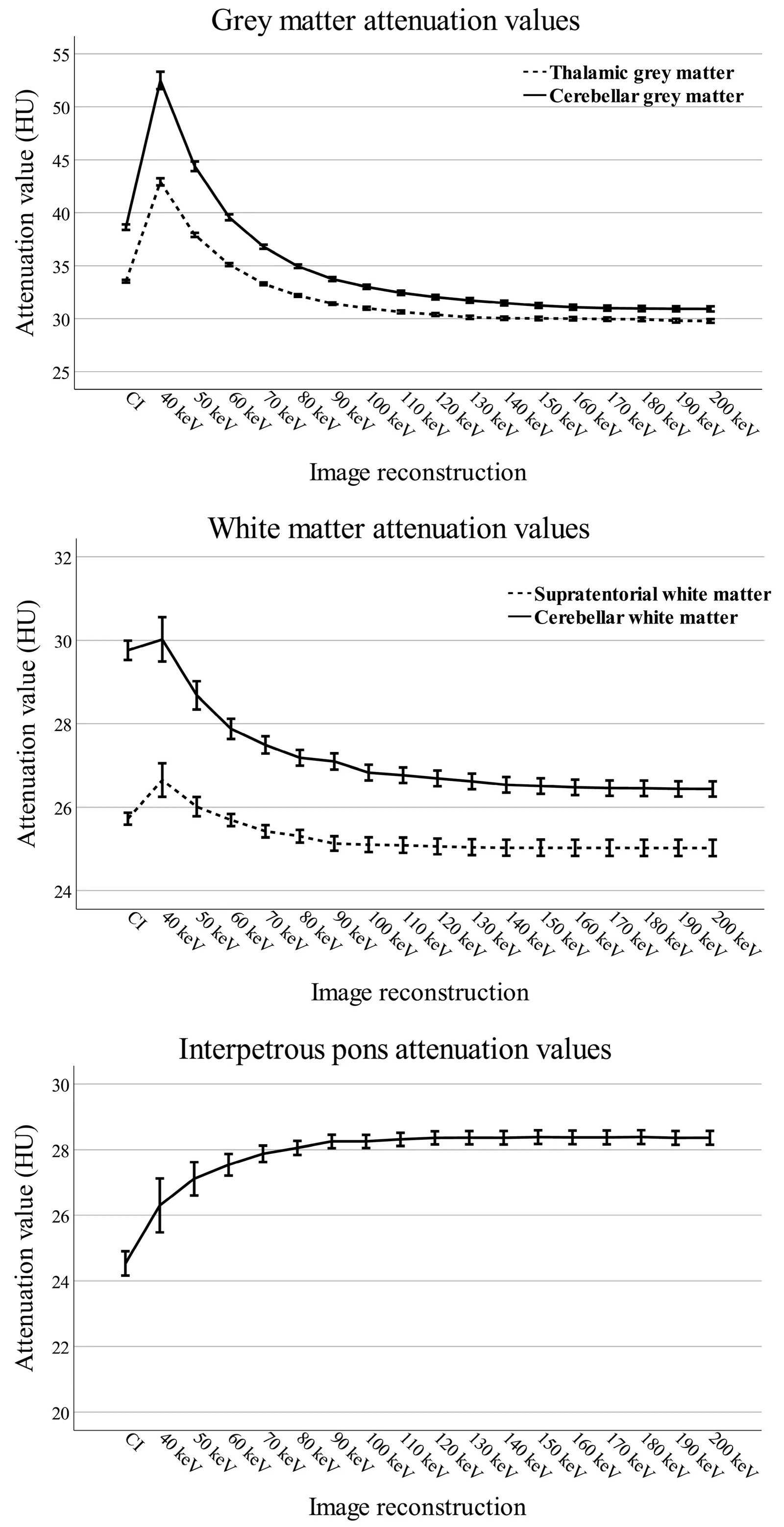
Mean attenuation values in supratentorial grey matter, supratentorial white matter, cerebellar grey matter, cerebellar white matter, and the interpetrous pons for conventional images (CIs) and virtual monoenergetic images (VMIs; 40–200 keV). Error bars represent 95% confidence intervals.

In the interpetrous pons, mean attenuation increased with increasing VMI energy and reached a plateau above approximately 100 keV. Attenuation was significantly higher in all VMIs than in CIs (Figure 2).

#### 3.2.2. Image Noise

Image noise was the highest in the 40 keV VMIs for cerebellar and reference grey matter (GM) and white matter (WM) and decreased with increasing VMI energy. Compared with CIs, image noise was significantly lower in VMIs ≥ 50 keV for supratentorial grey matter, supratentorial white matter, and cerebellar white matter. In cerebellar grey matter, image noise was significantly lower in VMIs ≥ 60 keV than in CIs.

#### 3.2.3. Posterior Fossa Artifact Index (PFAI)

**Figure 3:**
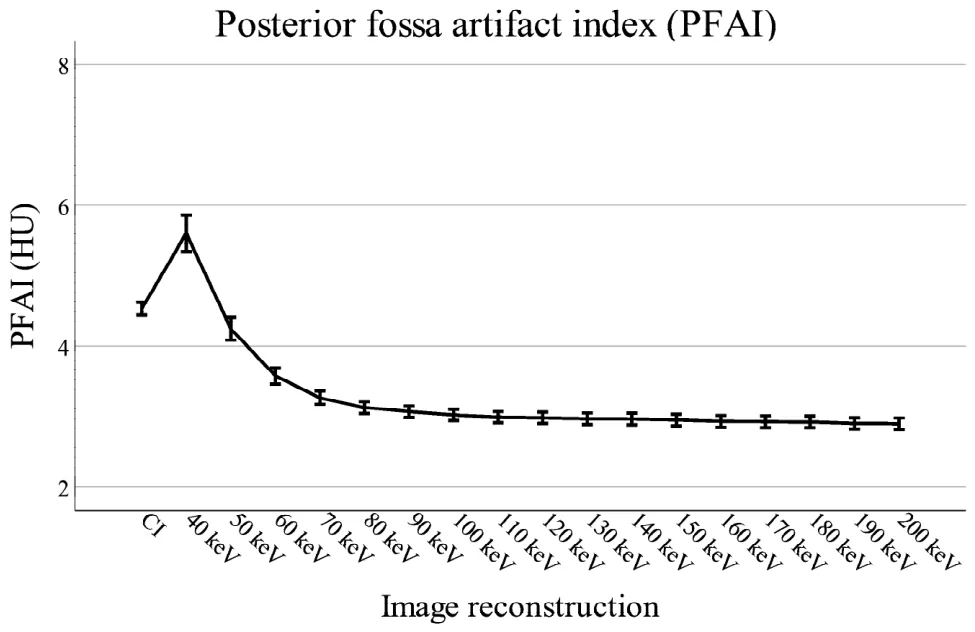
Posterior fossa artifact index (PFAI) in conventional images (CIs) and virtual monoenergetic images (VMIs; 40–200 keV). Error bars represent 95% confidence intervals.

The mean PFAI was 4.5 in CIs. In VMIs, PFAI was the highest at 40 keV (5.6) and decreased progressively with increasing energy, reaching a plateau at approximately 100 keV (Figure 3). Compared with CIs, PFAI was significantly lower in VMIs ≥ 50 keV. Significant differences between adjacent VMI energy levels were observed up to 110 keV.

#### 3.2.4. Signal-to-Noise and Contrast-to-Noise Ratios

The SNR was the lowest in the 40 keV VMIs for cerebellar grey matter, cerebellar white matter, and the interpetrous pons. The SNR increased progressively with increasing VMI energy and was significantly higher in VMIs ≥ 50 keV than in CIs for all ROIs.

**Figure 4:**
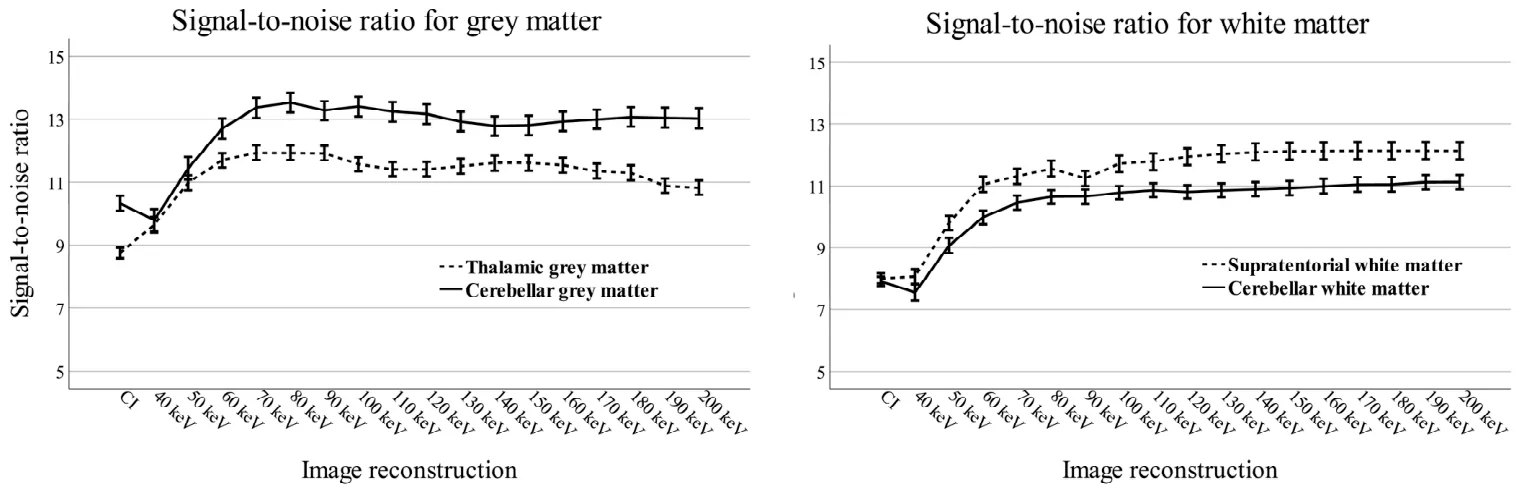
Signal-to-noise ratio (SNR) in conventional images (CIs) and virtual monoenergetic images (VMIs; 40–200 keV). Error bars represent 95% confidence intervals.

**Figure 5:**
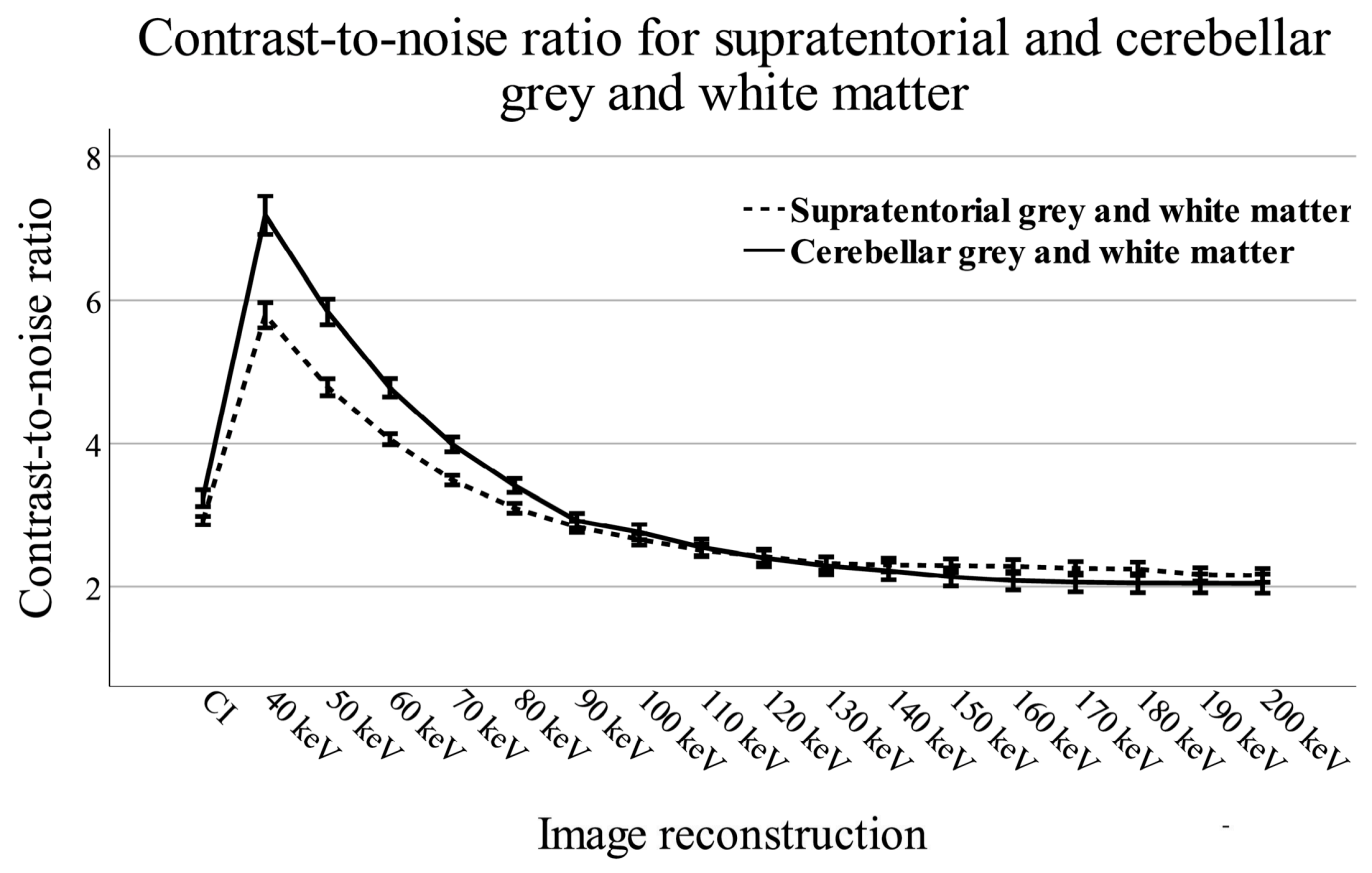
Contrast-to-noise ratio (CNR) in conventional images (CIs) and virtual monoenergetic images (VMIs; 40–200 keV). Error bars represent 95% confidence intervals.

The CNR was the highest at 40 keV and remained significantly higher than in CIs in VMIs between 40 and 80 keV for both supratentorial and cerebellar tissue. At ≥90 keV, the CNR decreased below that of CIs (Figure 4 and Figure 5).

#### 3.2.5. Difference in Attenuation

**Figure 6:**
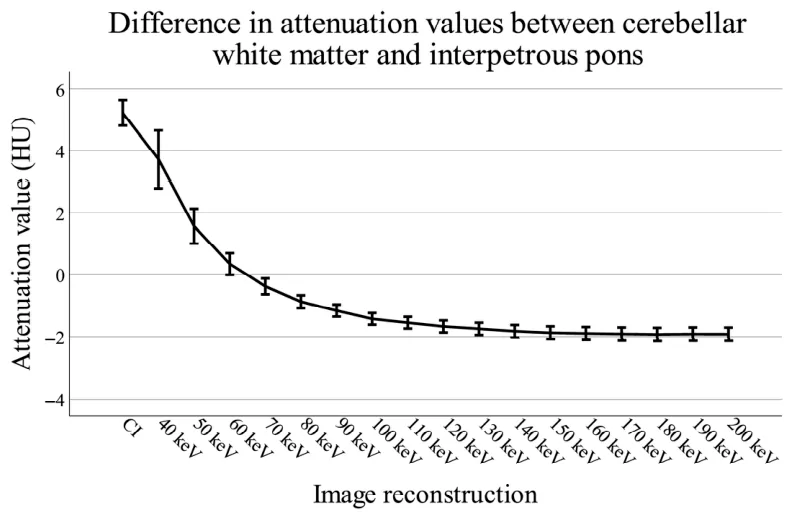
Mean attenuation difference (ΔHU) between cerebellar white matter and the interpetrous pons in conventional images (CIs) and virtual monoenergetic images (VMIs; 40–200 keV). Error bars represent 95% confidence intervals.

The mean attenuation difference (ΔHU) between cerebellar white matter and the interpetrous pons was the greatest in CIs (5.2 ± 2.9 HU). In VMIs, ΔHU decreased progressively with increasing energy and crossed zero between 60 and 70 keV. ΔHU was significantly lower in all VMIs than in CIs (Figure 6).

### 3.3. Qualitative Analysis

#### 3.3.1. Overall Image Quality

Reviewer 1 assigned CIs a mean overall image quality score of 2.4 (95% confidence interval [CI] 2.1–2.6). The highest mean score was observed for the 50 keV VMIs (4.3, 95% CI 4.1–4.5), and all VMIs between 40 and 80 keV received significantly higher scores than CIs (all *p* < 0.001).

**Figure 7:**
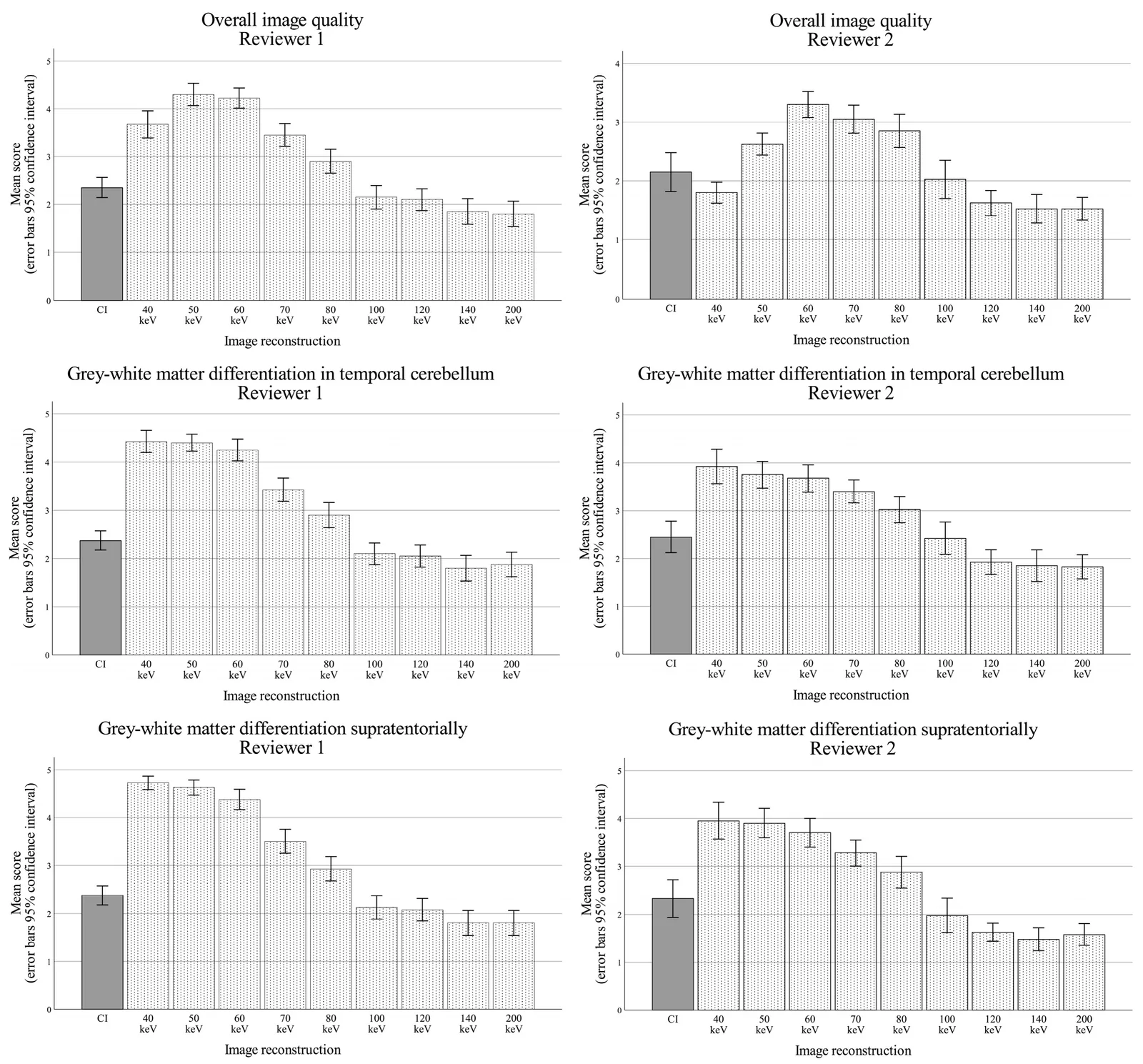
Mean qualitative scores for overall image quality (**top**), grey–white matter differentiation in the cerebellum (**middle**), and the supratentorial brain (**bottom**). Error bars represent 95% confidence intervals.

Reviewer 2 assigned CIs a mean score of 2.2 (95% CI 1.8–2.5). The highest mean score was observed for the 60 keV VMIs (3.3, 95% CI 3.1–3.5). VMIs between 50 and 80 keV all received significantly higher scores than CIs (Figure 7), with the largest improvement observed at 60 keV (*p* < 0.001).

#### 3.3.2. Grey–White Matter Differentiation

*Grey–white matter differentiation in the cerebellum*

Reviewer 1 assigned CIs a mean grey–white matter differentiation score of 2.4 (95% CI 2.2–2.6), while Reviewer 2 assigned a mean score of 2.5 (95% CI 2.1–2.8). For Reviewer 1, the highest mean score was observed at 40 keV (4.4, 95% CI 4.2–4.7), although the differences between 40, 50, and 60 keV were small. VMIs between 40 and 80 keV all received significantly higher scores than CIs (all *p* < 0.001).

Reviewer 2 likewise rated the 40 keV VMIs the highest (3.9, 95% CI 3.6–4.3). VMIs between 40 and 80 keV all received significantly higher scores than CIs (all *p* < 0.005).

*Grey–white matter differentiation in the cerebral hemispheres*

Reviewer 1 assigned CIs a mean grey–white matter differentiation score of 2.4 (95% CI 2.2–2.6). The highest mean score was observed at 40 keV (4.7, 95% CI 4.6–4.9), and all VMIs between 40 and 80 keV received significantly higher scores than CIs (all *p* < 0.001).

For Reviewer 2, the mean CIs score was 2.3 (95% CI 1.9–2.7). The highest mean score was likewise observed at 40 keV (4.0, 95% CI 3.6–4.3). VMIs between 40 and 70 keV all received significantly higher scores than CIs (all *p* < 0.001) (Figure 7).

#### 3.3.3. Artifact Severity

*Severity of artifacts in the cerebellum*

Reviewer 1 assigned CIs a mean artifact severity score of 2.9 (95% CI 2.8–3.0; higher scores indicate less severe artifacts). The highest mean score was observed at 60 keV (3.4, 95% CI 3.3–3.6), which was significantly higher than that of CIs (*p* < 0.001).

**Figure 8:**
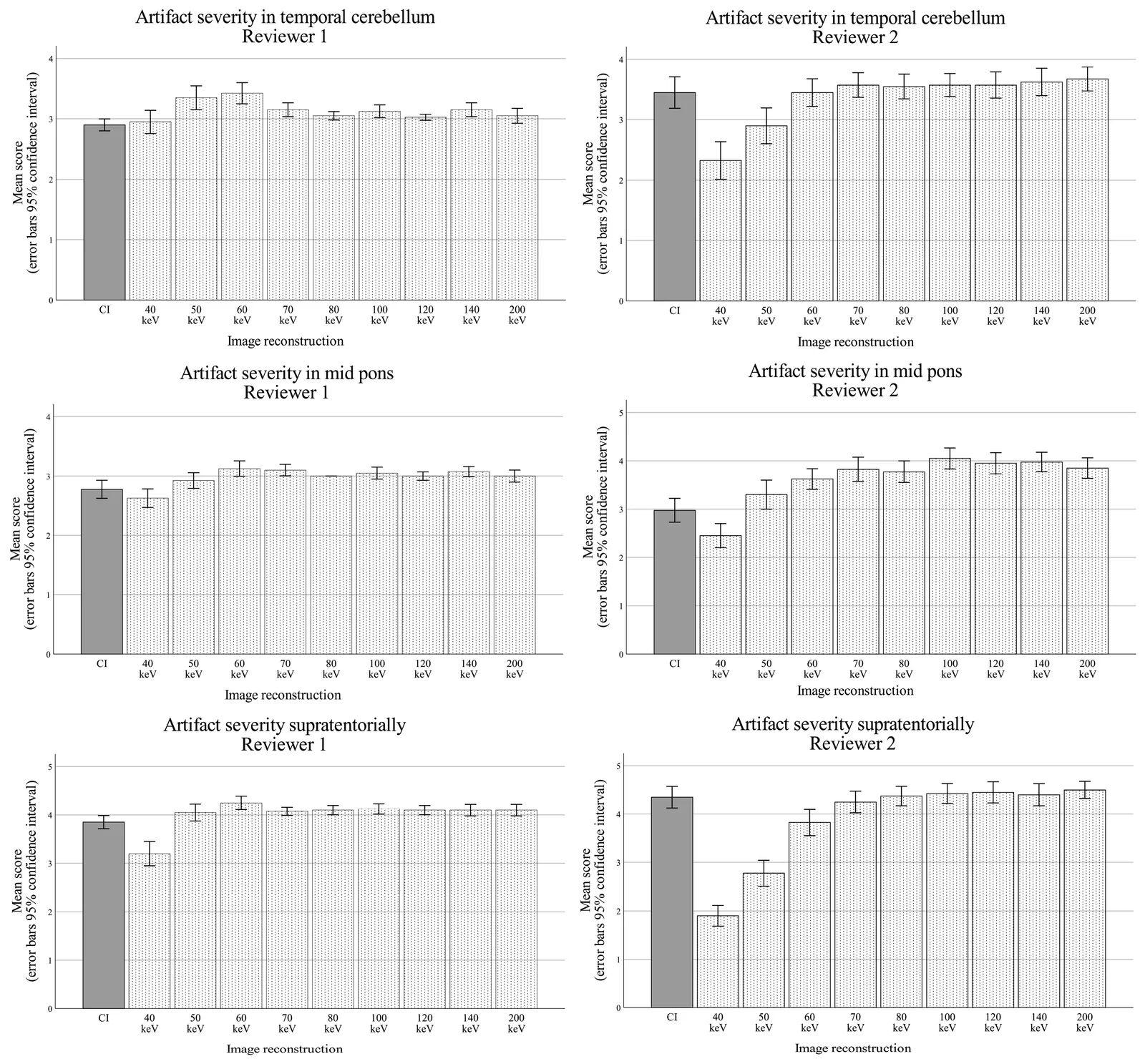
Mean artifact severity scores in the cerebellum (**top**), interpetrous pons (**middle**), and supratentorial brain (**bottom**) for both reviewers. Error bars represent 95% confidence intervals.

Reviewer 2 assigned CIs a mean score of 3.5 (95% CI 3.2–3.7). The highest mean score was observed at 200 keV (3.7, 95% CI 3.5–3.9), although differences between VMIs reconstructed at 60–200 keV were small. Compared with CIs, only the 40 and 50 keV VMIs received significantly lower scores (Figure 8).

*Severity of artifacts in the interpetrous pons*

Reviewer 1 assigned CIs a mean artifact severity score of 2.8 (95% CI 2.6–2.9). The highest mean score was observed at 60 keV (3.1), although differences between VMIs reconstructed at 50–200 keV were small. VMIs between 60 and 200 keV all received significantly higher scores than CIs (all *p* < 0.05).

**Figure 9:**
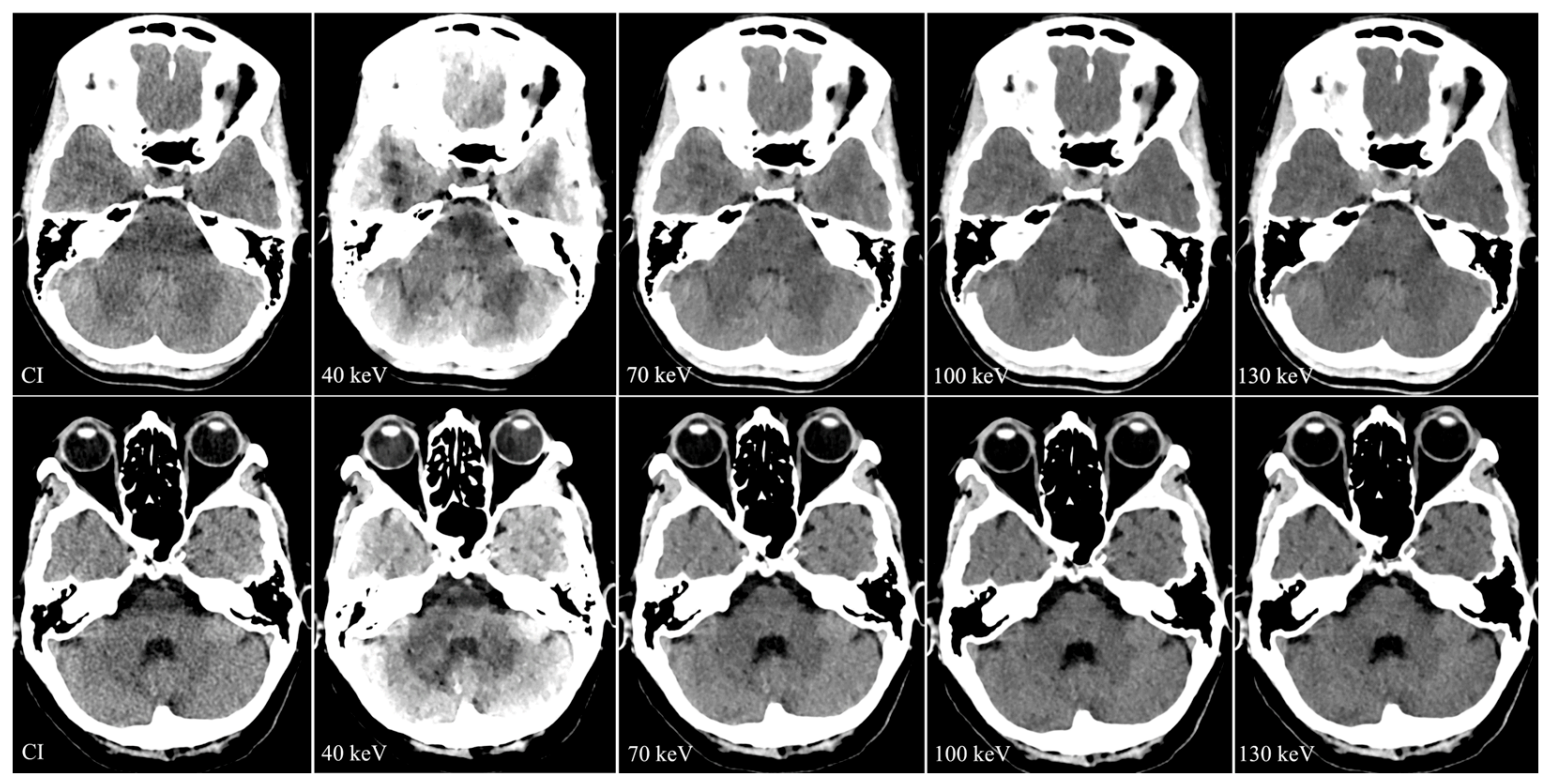
Representative conventional image (CI) and virtual monoenergetic images (VMIs) reconstructed at 40, 70, 100, and 130 keV from two patients, illustrating artifact reduction in the interpetrous pons with increasing VMI energy.

Reviewer 2 assigned CIs a mean score of 3.0 (95% CI 2.7–3.2). The highest mean score was observed at 100 keV (4.1, 95% CI 3.8–4.3). VMIs ≥ 50 keV all received significantly higher scores than CIs (all *p* < 0.05), whereas the 40 keV VMIs received significantly lower scores (*p* = 0.004) (Figure 8 and Figure 9).

*Severity of supratentorial subcalvarial artifacts*

Reviewer 1 assigned CIs a mean artifact severity score of 3.9 (95% CI 3.7–4.0). The highest mean score was observed at 60 keV (4.3, 95% CI 4.1–4.4), although differences between VMIs reconstructed at 50–200 keV were small. Only the 60 keV VMIs received significantly higher scores than CIs (*p* < 0.001).

Reviewer 2 assigned CIs a mean score of 4.4 (95% CI 4.1–4.6). The highest mean score was observed at 200 keV (4.5, 95% CI 4.3–4.7), although differences between VMIs reconstructed at 70–200 keV were minimal. No significant differences were observed between CIs and VMIs reconstructed at 70–200 keV, whereas VMIs at 40, 50, and 60 keV received significantly lower scores than CIs (all *p* < 0.05).

#### 3.3.4. Diagnostic Acceptability

Reviewer 1 considered 90% of CIs to be of sufficient image quality to exclude major posterior fossa pathology. Diagnostic acceptability was the highest for the 60 and 70 keV VMIs, for which all examinations were considered diagnostic. In contrast, the 140 keV VMIs had the highest proportion of non-diagnostic examinations (37.5%). For the supratentorial brain, 97.5% of CIs were considered diagnostic, while all examinations reconstructed at 50–70 keV were rated as diagnostically acceptable.

In contrast, Reviewer 2 considered only 37.5% of CIs to be of sufficient image quality to exclude major posterior fossa pathology. The proportion of non-diagnostic examinations was the lowest for the 60 keV (12.5%) and 70 keV (10%) VMIs. For the supratentorial brain, 42.5% of CIs were considered diagnostically acceptable, whereas the highest proportion of diagnostically acceptable examinations was observed for the 60 keV VMIs (92.5%).

#### 3.3.5. Intra- and Inter-Reader Agreement

Intra-reader agreement was assessed only for Reviewer 1 and was moderate for overall image quality (Cohen’s κ = 0.57) and poor for artifact severity in the cerebellum (κ = 0.19). Inter-reader agreement was fair for both overall image quality (κ = 0.32) and cerebellar artifact severity (κ = 0.22).

## 4. Discussion

This study demonstrates that virtual monoenergetic images (VMIs) reconstructed from dual-layer detector spectral CT substantially reduce posterior fossa beam-hardening artifacts while preserving, and in several respects improving, overall image quality compared with conventional images. Quantitative analyses showed that image noise and posterior fossa artifact severity decreased progressively with increasing VMI energy, whereas tissue attenuation and the contrast-to-noise ratio (CNR) were the highest at lower energy levels. Qualitative assessment by two neuroradiologists consistently identified VMIs reconstructed at approximately 50–70 keV as providing the best overall image quality. No single reconstruction energy was optimal for every image quality metric. Rather, lower-energy VMIs improved tissue contrast, whereas progressively higher energies reduced image noise and beam-hardening artifacts. Intermediate-energy reconstructions (approximately 50–70 keV) achieved the most favorable overall balance between these competing properties and therefore received the highest overall qualitative ratings.

The assessment of diagnostic adequacy should be interpreted cautiously because only examinations without intracranial pathology were included. Consequently, this endpoint reflects reader confidence in image quality rather than true diagnostic performance. Whether the observed improvements translate into improved lesion detection requires dedicated diagnostic accuracy studies including patients with posterior fossa pathology. The qualitative assessment demonstrated only fair inter-reader agreement for several subjective endpoints, reflecting the inherent subjectivity of image quality assessment and the absence of universally accepted criteria for grading posterior fossa artifacts. Importantly, despite differences in absolute scoring between readers, both observers consistently identified VMIs reconstructed between approximately 50 and 70 keV as providing the most favorable overall balance between artifact reduction and image quality. Thus, the principal qualitative conclusions were supported despite variability in individual scoring behavior. Taken together, these findings indicate that intermediate-energy VMIs achieve the most favorable balance between artifact suppression and preservation of tissue contrast.

Since all examinations were acquired on a single first-generation dual-layer detector spectral CT system using a standardized institutional protocol. Although the physical principles underlying monoenergetic reconstruction are common across spectral CT technologies, different implementations—including dual-source CT, rapid kV-switching systems, and photon-counting CT—differ in spectral separation, reconstruction methodology, and noise optimization. Consequently, while the overall trends observed in this study are likely to be broadly applicable, the precise reconstruction energy providing the optimal balance between artifact suppression, image noise, and tissue contrast may differ between vendors, scanner generations, and acquisition protocols. The present recommendation of approximately 50–70 keV should therefore be interpreted as specific to the investigated dual-layer detector platform until validated on other systems.

The quantitative results illustrate the fundamental trade-off inherent to monoenergetic CT reconstruction. Lower-energy VMIs increase tissue attenuation and improve tissue contrast through greater photoelectric absorption, thereby increasing CNR. However, these benefits are accompanied by increased image noise and more pronounced beam-hardening artifacts. Conversely, increasing the monoenergetic reconstruction energy progressively suppresses beam-hardening artifacts and reduces image noise but at the expense of tissue contrast. These findings are fully consistent with the physical principles underlying dual-energy CT and virtual monoenergetic image reconstruction [5,7].

Interestingly, the attenuation difference between cerebellar white matter and the interpetrous pons crossed zero between 60 and 70 keV. This observation reflects the opposing attenuation trends of normal brain tissue and beam-hardening artifacts with increasing monoenergetic energy and provides a quantitative explanation for why intermediate-energy VMIs achieved the highest qualitative ratings.

Our findings are consistent with previous studies demonstrating improved posterior fossa image quality using virtual monoenergetic reconstructions. Investigations using both dual-layer detector CT and rapid kVp-switching DECT have similarly reported reductions in beam-hardening artifacts and improvements in overall image quality compared with conventional images [8,9,10,16,17]. However, these studies generally included smaller patient cohorts and evaluated fewer monoenergetic reconstructions than the present study. Compared with previous studies, the present work combines the largest reported patient cohort with comprehensive quantitative measurements across the full clinically relevant monoenergetic spectrum, enabling characterization of how competing image-quality metrics together determine the optimal reconstruction energy.

The quantitative findings also closely paralleled previous observations. As reported by Neuhaus et al. and Pomerantz et al., the posterior fossa artifact index decreased rapidly with increasing monoenergetic energy before reaching a plateau above approximately 100 keV [8,9]. In contrast to Neuhaus et al., we observed significant differences between conventional images and VMIs, which likely reflects the greater statistical power afforded by our larger study population.

The attenuation difference between cerebellar white matter and the interpetrous pons crossed zero at slightly lower energies than previously reported by Zhao et al., who observed this transition between 75 and 80 keV [10]. Given the relatively small attenuation differences involved, together with differences in ROI placement, reconstruction parameters, and post-processing software, these findings are nevertheless highly consistent.

From a clinical perspective, the present findings suggest that routine reconstruction of VMIs at approximately 60 keV may improve evaluation of the posterior fossa without compromising supratentorial image quality. This may be particularly relevant in emergency neuroradiology, where beam-hardening artifacts may obscure small hemorrhages or early posterior circulation infarction and MRI is often unavailable or delayed [3,4].

Although our study assessed image quality rather than diagnostic accuracy, the qualitative assessments indicate that intermediate-energy VMIs may increase diagnostic confidence during routine evaluation of the posterior fossa.

Diagnostic performance cannot be established from a cohort consisting exclusively of normal examinations. Nevertheless, the improved image quality observed in the present study is consistent with previous work suggesting improved detection of posterior fossa infarction using VMIs. Hixon et al. reported higher sensitivity for posterior circulation infarction using VMIs than conventional images, although the difference did not reach statistical significance, likely because of the relatively small study population [18]. Similar improvements have also been shown by using iterative reconstruction algorithms [19].

### Strengths and Limitations

The principal strengths of this study are its comparatively large cohort, comprehensive quantitative evaluation across the full monoenergetic spectrum, and independent qualitative assessment by two experienced neuroradiologists. Together, these enabled evaluation of not only individual image-quality metrics but also the balance between artifact suppression and preservation of tissue contrast that determines overall diagnostic image quality.

There are several limitations of this study.

First, this was a retrospective single-center study performed using a standardized imaging protocol at one tertiary referral center. Although this ensured a homogeneous study population and minimized technical variability, it may limit the generalizability of the findings to institutions using different patient populations, imaging protocols, or scanner settings. Prospective multicenter studies would be valuable to confirm the optimal reconstruction energy across a broader range of clinical settings.

Second, only examinations without intracranial pathology were included. This design allowed for evaluation of reconstruction-dependent image quality without confounding from lesion-related attenuation changes but precludes assessment of diagnostic accuracy. Consequently, although VMIs improved objective and subjective image quality, the present study cannot determine whether these improvements translate into improved detection of posterior fossa infarction, hemorrhage, tumors, or other pathological conditions. Future studies should evaluate diagnostic performance in clinically relevant patient cohorts.

Third, qualitative image assessment was based on subjective Likert-scale ratings. Although this reflects routine clinical image interpretation, subjective image-quality assessment inevitably introduces reader variability. Indeed, the two reviewers differed considerably in their absolute scoring, particularly regarding diagnostic acceptability, despite demonstrating similar trends across reconstruction energies. This suggests that the observed differences primarily reflect individual thresholds for image quality rather than disagreement regarding the optimal reconstruction energy.

Fourth, the study involved multiple comparisons across reconstruction energies and imaging metrics without formal adjustment for multiplicity. Although this increases the possibility of type I error, the principal conclusions were supported by consistent quantitative and qualitative findings across multiple independent image-quality measures.

Fifth, ROI placement was performed manually on the conventional images before automatic propagation to all monoenergetic reconstructions. Although this ensured identical ROI locations across reconstruction energies within each examination, reproducibility of the initial ROI placement was not formally evaluated and may have contributed to measurement variability.

Finally, all examinations were performed on a single dual-layer detector CT platform. Although the physical principles of virtual monoenergetic imaging are shared across DECT technologies, the optimal reconstruction energy may vary somewhat between vendors and reconstruction algorithms. The examinations were acquired using a first-generation dual-layer spectral CT platform and reconstructed using the software version available at the time of the study. Subsequent advances in reconstruction algorithms, iterative reconstruction techniques, and spectral post-processing may alter image noise characteristics and potentially shift the precise reconstruction energy providing the optimal balance between noise and artifact reduction. Nevertheless, the fundamental relationship between monoenergetic reconstruction energy and beam-hardening artifact suppression is expected to remain similar, although confirmation on newer software generations would be valuable.

## 5. Conclusions

Virtual monoenergetic images reconstructed at approximately 60 keV provide the most favorable balance between posterior fossa artifact reduction and preservation of image quality. Compared with conventional images, they improve both quantitative and qualitative image-quality metrics and can facilitate assessment of the posterior fossa in routine clinical practice.

Prospective studies including patients with posterior fossa pathology are warranted to determine whether these improvements translate into improved diagnostic performance.

## Data Availability

Requests to access an anonymized dataset supporting the conclusions of this article may be sent to the corresponding author after obtaining the appropriate ethics approval, due to privacy concerns and ethical restrictions.

## Abbreviations

The following abbreviations are used in this manuscript:

CI	Conventional polyenergetic Images
CNR	Contrast-to-Noise Ratio
CT	Computed Tomography
DECT	Dual Energy CT
DLP	Dose Length Product
GM	Grey Matter
HU	Hounsfield Units
keV	Kiloelectron Volt
MRI	Magnetic Resonance Imaging
PFAI	Posterior Fossa Artifact Index
ROI	Region of Interest
SD	Standard Deviation
SNR	Signal-to-Noise Ratio
VMI	Virtual Monoenergetic Images
WM	White Matter
